# Minimizing Complications of Aging That Lead to Dry Mouth and Poor Oral Health

**DOI:** 10.1093/geroni/igab046.966

**Published:** 2021-12-17

**Authors:** Athena Papas

**Affiliations:** Tufts University School of Dental Medicine, Boston, Massachusetts, United States

## Abstract

Poor oral health causes severe pain and untreated infections to spread throughout the body. For older adults, the prevalence of root decay exceeds that of any other medical condition. Our research shows tooth loss and edentulousness were associated with increased mortality and inversely associated with BMI, waist circumference, blood pressure, and fasting blood glucose. Our Stop-it study found people who lost bone density had fewer teeth, problems chewing, and involuntary weight loss and frailty. 88% of the elderly take medications that cause loss of saliva. Sjögren’s and radiation therapy for head and neck cancer patients heighten risk. Without saliva, patients have increased tooth decay, periodontal disease and fungal infections, salivary gland blockage, and problems swallowing and speaking. Dry mouth leads people to suck on candy that further increase caries. Substituting sugarfree gum for candy increases salivary flow and reduces dental caries. Brushing, flossing, and limiting sugar also lessen tooth decay.

